# Tumor taxonomy for the developmental lineage classification of neoplasms

**DOI:** 10.1186/1471-2407-4-88

**Published:** 2004-11-30

**Authors:** Jules J Berman

**Affiliations:** 1 Cancer Diagnosis Program, National Cancer Institute, Bethesda, USA

## Abstract

**Background:**

The new "Developmental lineage classification of neoplasms" was described in a prior publication. The classification is simple (the entire hierarchy is described with just 39 classifiers), comprehensive (providing a place for every tumor of man), and consistent with recent attempts to characterize tumors by cytogenetic and molecular features. A taxonomy is a list of the instances that populate a classification. The taxonomy of neoplasia attempts to list every known term for every known tumor of man.

**Methods:**

The taxonomy provides each concept with a unique code and groups synonymous terms under the same concept. A Perl script validated successive drafts of the taxonomy ensuring that: 1) each term occurs only once in the taxonomy; 2) each term occurs in only one tumor class; 3) each concept code occurs in one and only one hierarchical position in the classification; and 4) the file containing the classification and taxonomy is a well-formed XML (eXtensible Markup Language) document.

**Results:**

The taxonomy currently contains 122,632 different terms encompassing 5,376 neoplasm concepts. Each concept has, on average, 23 synonyms. The taxonomy populates "The developmental lineage classification of neoplasms," and is available as an XML file, currently 9+ Megabytes in length. A representation of the classification/taxonomy listing each term followed by its code, followed by its full ancestry, is available as a flat-file, 19+ Megabytes in length.

The taxonomy is the largest nomenclature of neoplasms, with more than twice the number of neoplasm names found in other medical nomenclatures, including the 2004 version of the Unified Medical Language System, the Systematized Nomenclature of Medicine Clinical Terminology, the National Cancer Institute's Thesaurus, and the International Classification of Diseases Oncolology version.

**Conclusions:**

This manuscript describes a comprehensive taxonomy of neoplasia that collects synonymous terms under a unique code number and assigns each tumor to a single class within the tumor hierarchy. The entire classification and taxonomy are available as open access files (in XML and flat-file formats) with this article.

## Background

On March 19, 2004, the author published a new classification for neoplasms, now called "The developmental lineage classification of neoplasms"[1]. The classification was described as a schema providing a single class location for every tumor in man. The classification contains 39 class descriptors appearing as XML tags.

Good classifications encapsulate all information relating to a knowledge domain. Modern classifications allow us to understand complex entities by grouping them by their shared or inherited properties [2]. Modern classifications also allow us to retrieve and integrate many different kinds of data under a common conceptual framework [3-8]. Basic to every classification is a taxonomy, the complete listing of the instances of the knowledge domain [2]. Because computers make it easy to store, organize and retrieve any number of listed items, there is no reason to limit taxonomies to a small number of preferred terms. One of the the best examples of a large taxonomy is taxonomy.dat, which attempts to list every living organism on earth [9]. So thorough is taxonomy.dat that it not only lists all known variations of an organism's name, it also lists commonly used misspellings of an organism. An example of an entry in taxonomy.dat:

ID : 50

PARENT ID : 49

RANK: genus

GC ID : 11

SCIENTIFIC NAME : Chondromyces

SYNONYM : Polycephalum

SYNONYM : Myxobotrys

SYNONYM : Chondromyces Berkeley and Curtis 1874

SYNONYM : "Polycephalum" Kalchbrenner and Cooke 1880

SYNONYM : "Myxobotrys" Zukal 1896

MISSPELLING : Chrondromyces

A recent version of taxonomy.dat is dated June 20, 2004 and is 55,233,858 bytes in length. It has 246,800 entries The taxonomy.dat file is available for public download through anonymous ftp [9]. The creation and organization of biological information is one of the most active areas of biomedical research [10,11].

The taxonomy for the developmental lineage classification of neoplasms was loosely modeled on taxonomy.dat. An attempt was made to include the name of every tumor, including every known variant of tumor name, and to assign a unique numeric code to all synonyms for a given tumor. The purposes of this paper are: 1) to publicly release the neoplasm taxonomy database file; 2) to explain its role as the source of concept instances for the developmental lineage classification of neoplasms; 3) to describe the methods used to organize the taxonomy; and 4) to compare the taxonomy with the neoplasm nomenclature contained in the Unified Medical Language System Metathesaurus (UMLS), the largest medical nomenclature in existence, and with the Systematized Nomenclature of Medicine Clinical Terminology (SNOMED-CT) [see Additional file 1] [see Additional file 2][12,13].

## Methods

The developmental lineage classification of neoplasms was described in a prior publication [1]. The classification was intended to be populated by a comprehensive taxonomy. The original publication contained a relatively short first-draft taxonomy, and the current taxonomy was built on the early draft [1]. To ensure compatibility between the classification and other biomedical databases that include neoplasm terms, terms and classes were formatted in XML [14,15].

Terms were grouped by concept and examined for completeness, with software written in the Perl programming language. Perl is a free, open source language, available for virtually every computer operating system, and widely used in the bioinformaitcs community [16,17]. Nomeclature terms often have alternate forms that can be discovered and accrued with the help of software [18]. Short Perl scripts were prepared to systematically add variant names for patterned term constructs. For instance, it was noticed that some terms appeared in the form of "adenocarcinoma of [organ]" and other terms appeared as "adenocarcinoma of the [organ]." A Perl script ensured that both forms were included for this and other examples.

As the taxonomy enlarged, inadvertent duplications of terms and codes were unavoidable. In addition, duplicate terms were occasionally placed into different classes within the hierarchy. Much of the value of a classification comes from the parsimonious deployment of taxons (i.e. no instance can appear in more than one class). A Perl script was prepared that was executed after each modification to the classification/taxonomy. The Perl script parsed through the updated XML classification/taxonomy file, validating that: 1) each term occurs only once in the taxonomy; 2) each term occurs in only one tumor class; 3) each concept code occurs in only one hierarchical position in the classification; and 4) the file containing the classification and taxonomy is a well-formed XML document. The script finds anomalous records, permitting facile repair of the taxonomy file. The validating Perl script, xmlvocab.pl is included as a supplemental file with this manuscript [see Additional file 3]

A perl script transformed the taxonomy XML file into a flat-file consisting of line-records for each term in the taxonomy (neoself.txt, 19+ Megabytes in length). The transforming Perl script is neoself.pl and is distributed with this article [see Additional file 4].

The taxonomy was compared with the neoplasm terms and concepts included in the UMLS [12]. UMLS is produced and curated by the U.S. National Library of Medicine. UMLS concepts and terms are drawn from over 100 different medical source vocabularies. It is the largest medical nomenclature in existence. The 2004 version of the UMLS Metathesaurus used in this study includes over 2,697,491 medical terms. This version of the UMLS is the first UMLS version to contain the Systematized Nomenclature of Medicine – Clinical terminology (SNOMED-CT) [13]. As in prior versions, the 2004 UMLS contains the National Cancer Institute Thesaurus, another rich source of neoplasm terminology [6]. The International Classification of Diseases – Oncology (ICD-O), is a nomenclature prepared by the World Health Organization [19]. Although the UMLS does not list the ICD-O as a contributing thesaurus, it can be noted that ICD-O terminology is incorporated into the SNOMED-CT nomenclature included in UMLS [20].

The UMLS is curated and distributed by the U.S. National Library of Medicine [12]. Although the UMLS is publicly available, there are numerous restrictions on its use, and those wishing to download the UMLS must enter into a license agreement with the National Library of Medicine before obtaining the Metathesaurus.

All terms from the UMLS Metathesaurus that have a neoplasm relationship were obtained through the use of a Perl script [see Additional file 5]. The 2004 UMLS files used for the extraction were:

MRCON (UMLS metathesaurus file, 2004 version, 198,586,537 bytes in length), containing the terms for each UMLS concept (CUI).

MRCXT (UMLS metathesaurus file, 2004 version, 8,347,732,946 bytes in length), containing the relationships for every UMLS code.

All MRCXT records with a SNOMED-CT derivation were extracted using a Perl script [see Additional file 6]. All MRCON records containing a neoplasm term and having a SNOMED-CT origin were extracted using another Perl script [see Additional file 7].

## Results and discussion

### Features of the taxonomy

The taxonomy currently contains 122,632 different terms encompassing 5,376 neoplasm concepts (neocl.xml). Each concept has, on average, 23 synonymous terms. The second-largest source of tumor names is contained in the licensed 2004 version of the Unified Medical Language System Metathesaurus (UMLS), which draws neoplasm terms from the National Cancer Institute Thesaurus and SNOMED-CT. SNOMED-CT incorporates the International Classification of Disease – Oncology) [6,12,13,19,20].

The UMLS contains 24,593 unique English neoplasm terms with a specific "neoplasms" relationship, about one fifth the number of terms contained in the taxonomy. However, when one counts the terms in UMLS that have ANY type of relationship to neoplasia, the number UMLS-derived neoplasia terms expands to 64,601, about half of the number of terms contained in the taxonomy. It is difficult, if not impossible, to determine a correct number of neoplasm names contained in UMLS. The reason is that in UMLS, a concept may have many different relationships. For instance, the UMLS concept for "abdominal pain" has 805 relationships. Among these are: colic, constipation, diarrhea, influenza-like symptoms, malaise, multiple organ failure AND GI neoplasm benign, GI neoplasm malignant. The last two items are relationships to neoplasms. These relationships are valid because abdominal pain can be associated with benign or malignant neoplasms. Although the Perl script ca_mrrec.pl [see Additional file 5] outputs over 64,000 terms with neoplasm relationships, many of these terms are not names of neoplasms. If all 64.601 terms were reviewed to determine which were valid tumor names, a subjective number would be obtained that would certainly differ with each reviewer. It is probably fair to say that the number of UMLS neoplasm terms is somewhere between 24,593 (terms with a specific "neoplasms" relationship) and 64,601 (terms with any kind of relationship to neoplasms).

Similarly, the number of SNOMED-CT terms with a neoplasm relationship is 35,920. This is the number of UMLS records with a SNOMED-CT derivation and with any type of neoplasm relationship [see Additional file 7]. This should be considered an upper limit estimate and is less than a third of the number of neoplasm terms included in the taxonomy.

In general, taxonomy concepts that were highly generic (such as adenocarcinoma of lung) and the concepts that were the most highly pre-coordinated (i.e. multi-word terms with modifiers) had the greatest numbers of synonymous representations.

For example, consider these 48 synonyms for adenocarcinoma of the lung:

adenoca arising from lung, adenoca arising in lung, adenoca of lung, adenocarcinoma arising from lung, adenocarcinoma arising from pulmonary, adenocarcinoma arising from the lung, adenocarcinoma arising from the lungs, adenocarcinoma arising in lung, adenocarcinoma arising in pulmonary, adenocarcinoma arising in the lung, adenocarcinoma arising in the lungs, adenocarcinoma of lung, adenocarcinoma of pulmonary, ca arising from lung, ca arising from lungs, ca arising in lung, ca arising in lungs, ca of lung, ca of lungs, cancer arising from lung, cancer arising from lungs, cancer arising in lung, cancer arising in lungs, cancer of lung, cancer of lungs, carcinoma arising from lung, carcinoma arising from lungs, carcinoma arising in lung, carcinoma arising in lungs, carcinoma of lung, carcinoma of lungs, lung adenoca, lung adenocarcinoma, lung ca, lung cancer, lung with adenoca, lung with adenocarcinoma, lung with ca, lung with cancer, lung with carcinoma, lungs with ca, lungs with cancer, lungs with carcinoma, pulmonary adenoca, pulmonary adenocarcinoma, pulmonary ca, pulmonary cancer, pulmonary carcinoma

Commonly occurring lesions have many different representations. The taxonomy contains closely-related but non-synonymous concepts as separate entries (e.g. there are 29 synonyms for bronchogenic carcinoma, and 15 synonyms for bronchioloalveolar adenocarcinoma)

Other items in the taxonomy that have multiple term-variants are the so-called pre-coordinated terms characterized by modifying phrases. These terms are the hardest to capture in a taxonomy, and seem to return the smallest value on the effort. For instance, the taxonomy contains 118 synonyms for "testis with mixed embryonal carcinoma and endodermal sinus neoplasm with seminoma." The large number of synonyms are the direct result of the many different ways that modifying phrases can be ordered and combined to create the same terms. A few examples of the 118 synonyms for this term are:

mixed embryonal cancer and endodermal sinus neoplasm with seminoma arising in testis

mixed embryonal cancer and endodermal sinus neoplasm with seminoma of testis

testis with mixed embryonal cancer and endodermal sinus tumor with seminoma

mixed embryonal cancer and endodermal sinus tumor with seminoma arising in testis

testis with mixed embryonal cancer and yolk sac neoplasm with seminoma

mixed embryonal cancer and yolk sac neoplasm with seminoma arising in testis

mixed embryonal cancer and yolk sac neoplasm with seminoma arising from testis

mixed embryonal cancer and yolk sac neoplasm with seminoma of testis

testis with mixed embryonal cancer and yolk sac tumor with seminoma

The taxonomy database is distributed as an XML or as a flat-file. A short excerpt from the XML file is shown:

<mesoderm>

<name nci-code = "C3731000">mesoblastic nephroma</name>

<name nci-code = "C3731100">cellular mesoblastic nephroma</name>

<mesoderm> indicates a class tag. Beneath it are two different terms and concepts. Each is given a unique concept number. The second term is similar to the first term, but not identical. Both code numbers share the first 4 digits.

The flat-file version of the taxonomy lists these two terms as line-records. Each line record contains the term name, the term code, and the ancestry of the term within the developmental lineage classification. Each ancestor is separated by its predecessor by an arrow character.

mesoblastic nephroma|C3731000|mesoderm>non_primitive>

embryonic>neoplasms>tumor_classification>

cellular mesoblastic nephroma|C3731100|mesoderm>non_primitive>

embryonic>neoplasms>tumor_classification>

Medical informaticians dream of the day when all medical data will be captured by computers in a highly structured format that ensures data uniformity. In this utopian vision, only canonical forms of medical terms will be used. Medical reports will have a uniform format, and will be computer parsable and human readable. Taxonomies will be small. Unfortunately, the current trend in medical reporting seems to favor unstructured narrative data entry. Personally, I can remember the early days of computers when data storage and memory constraints were at a premium. Years were entered as two-digit values (nobody worried about Y2K back in the 60s), and entry-words were selected from lists and typically represented by a single digit. Today, the storage and transmission of textual data are non-issues. Large vocabularies of millions of terms can reside in active memory. Physicians prefer narrative text over structured text [21], and most of the medical data entered by physicians appears in the form of free-text emails, memoranda, progress notes, hospital reports of every type, research publications, etc. Free expression results in a seemingly unlimited way of describing a single thought, and large taxonomies are sometimes useful tools for organizing and retrieving the many terms found in narrative free-text.

The neoplasm taxonomy was created to collect all ways of expressing the names of all human tumors. The purpose of the taxonomy is to make it possible for computer algorithms to index textual information about all tumors regardless of the terms used to describe particular tumors. At first inspection, this may seem like a hopelessly complex and ultimately futile endeavor. Can anyone seriously hope to make sense of narrative text? Won't the taxonomy become larger and more complex as additional clinical, genomic, and proteomic modifiers split tumors into incomprehensible subcategories?

Actually, the purpose of a taxonomy is to reduce the complexity of the knowledge domain. By focusing efforts on a relatively small area of medicine, it is feasible to create a product that does not exceed the limits of a single expert's mental capacity. The large number of terms contained in the taxonomy (122,632), is encompassed by just 5,376 concepts. We can parse through text, replacing the many different variants of a term with either a single concept code or with a preferred (so-called canonical) synonym. This means that the taxonomy gives us a method of reducing any document index of neoplastic terms down to a maximum of 5,376 canonical terms. In addition, the 5,376 concepts in the taxonomy are represented by 39 different ancestral classes. The developmental lineage classification of neoplasms is constructed using strict rules: no multi-class inheritance; each subclass endowed by the properties of its ancestor. This means that once we have identified a term, we can easily determine its place among just a few dozen classes, and its ancestry should tell us basic information about the biology of the tumor [1].

The taxonomy for the developmental lineage classification of neoplams is the largest neoplasm taxonomy. It should come as no surprise that large nomenclatures provide better coverage of textual terms than smaller nomenclatures. A 1997 study by Humphreys et al., showed that by combining controlled vocabularies, the UMLS provided substantially more exact matches to free-text terms than any individual vocabulary in the nomenclature [22]. Finding new ways of expanding terminologies is an active research area [18]. The current taxonomy is large and has benefited from the use of Perl scripts that validate the database for internal sense and consistency.

## Conclusions

This manuscript describes a comprehensive taxonomy of neoplasia that collects synonymous terms under a unique code number and assigns each tumor to a single class within a tumor hierarchy. The entire classification and taxonomy are available as open access documents (in XML and flat-file formats) with this article. The taxonomy will be merged into the U.S. National Cancer Institute's Thesaurus, a curated, publicly available nomenclature and ontology that includes neoplasms and cancer-related terminology.

## Competing interests

The author(s) declare that they have no competing interests.

## Authors' contributions

This work represents the opinions of the author and does not represent the policy of the NIH or of any other U.S. Federal Agency.

## Pre-publication history

The pre-publication history for this paper can be accessed here:



## Supplementary Material

Additional File 1 **Neoplasia classification structure (XML version)** Neoclxml.gz is a compressed (gzipped) XML file. The downloaded file should be renamed neoclxml.gz so that the .gz suffix can be recognized by unzip utilities. Unzip the file (using a free, open source utility such as gunzip.exe [23], or a proprietary utility such as Winzip). Once unzipped, the file should be renamed neocl.xml, so that it will have an .xml suffix. If the file is too large for viewing on your web browser, it can be viewed on plain-text word processors. Click here for file

Additional File 2 **Neoplasia classification with taxonomy (flat-file plain-text version)** Neoself.gz is a compressed (gzipped) ascii flat-file. If the filename is changed during download, it should be renamed neoself.gz so that the .gz suffix can be recognized by unzip utilities. Unzip the file (using an open source utility such as gunzip.exe [23], or a proprietary utility such as Winzip). Once unzipped, the file is 19+ Mbytes in length. The expanded file should be renamed neoself.txt. Click here for file

Additional File 3 **Taxonomy validating Perl script** The validating Perl script is xmlvocab.pl. Perl scripts will execute on any computer with a Perl interpreter. It requires the external taxonomy file named "neocl.xml" residing in the same subdirectory as xmlvocab.pl. Click here for file

Additional File 4 **Perl script for transforming taxonomy XML file to a plain-text flat file** The Perl script neoself.pl transforms the XML database file (neocl.xml) to a flat file (neoself.txt). Click here for file

Additional File 5 **Perl script for extracting neoplasm codes and terms from UMLS** The Perl script ca_mrrec.pl produces a file (neomrcxt.txt) containing all UMLS codes and terms with a neoplasm relationship. This script requires the external files MRCXT and MRCON (available at no cost from the National Library of Medicine) to reside in the same directory as ca_mrrec.pl. This script may take more than one-half hour to execute. Click here for file

Additional File 6 **Perl script for extracting UMLS codes for SNOMED-derived terms** The Perl script snomout.pl produces a file (snomout.txt) containing all UMLS terms with a SNOMED-CT vocabulary relationship It requires the external file MRCXT (available at no cost from the National Library of Medicine) to reside in the same directory as snomout.txt. It produces an output file, snomout.txt that has a size of 1,895,054,040 bytes. This script may take more than one-half hour to execute. Click here for file

Additional File 7 **Perl script for extracting neoplasm concepts and terms from the SNOMED-CT subset of UMLS** The Perl script ca_snrec.pl produces a file (neosnom.txt) containing all UMLS terms derived from SNOMED-CT having a neoplasm relationship. It requires the external file MRCON (available at no cost from the National Library of Medicine) and snomout.txt (produced by snomout.pl) [see Additional file 6], both residing in the same directory as ca_snrec.pl. This script may take more than one-half hour to execute. Click here for file
